# Silencing Phytoene Desaturase Causes Alteration in Monoterpene Volatiles Belonging to the Methylerythritol Phosphate Pathway

**DOI:** 10.3390/plants11030276

**Published:** 2022-01-20

**Authors:** Nabil Killiny

**Affiliations:** Citrus Research and Education Center, Department of Plant Pathology, IFAS, University of Florida, 700 Experiment Station Road, Lake Alfred, FL 33850, USA; nabilkilliny@ufl.edu; Tel.: +1-863-956-8833

**Keywords:** phytoene desaturase, photobleaching, virus-induced gene silencing, *Citrus tristeza virus*, volatile organic compounds, methylerythritol phosphate, monoterpenes

## Abstract

Volatile organic compounds (VOCs) are a large group of lipophilic hydrocarbon compounds derived from different biosynthetic pathways in plants. VOCs are produced and released from plants as a defense mechanism against biotic and abiotic stresses. They are involved in communication with the surrounding environment including plant-to-plant interactions and attracting or repelling insects. In citrus, phytoene desaturase (PDS), a precursor of the carotenoid biosynthetic pathway has been silenced using the *Citrus tristeza virus*-induced gene silencing technique. Silencing *PDS* resulted in a reduction of carotenoid contents and in the photobleaching phenotype in leaves. Interestingly, the strength of the phenotype was varied within the plants due to the unequal distribution of virus particles. Using solid-phase microextraction (SPME), fibers released VOCs from leaves with gradient degrees of the photobleaching phenotype were collected and analyzed in gas chromatography-mass spectrophotometry (GC-MS). Overall, 47 VOCs belonging to 12 chemically distinguished groups were detected and identified using authentic standards. Simple linear regression showed that monoterpenes belonging to methylerythritol phosphate (MEP) were significantly corrected with the degrees of photobleaching (carotenoid content). Both carotenoids and MEP biosynthetic pathways occurred in the plastid. Thus, we provide preliminary evidence for a potential role of carotenoids in supporting the MEP pathway and/or the production of monoterpenes.

## 1. Introduction

Virus-induced gene silencing (VIGS) in *Citrus* spp. has been developed as a powerful tool to study functional genomics and to defend citrus against “*Ca*. Liberibacter asiaticus”, the pathogen associated with citrus Huanglongbing (HLB), also called citrus greening disease [1,2]. VIGS is achieved by using the mild strain T-36 of *Citrus tristeza virus* (CTV), a member of the genus *Closterovirus*, to knockdown endogenous genes in citrus tissues. Among the genes targeted through RNAi is the reporter gene phytoene desaturase (PDS), a key plant enzyme involved in carotenoid biosynthesis [3]. PDS facilitates the conversion of phytoene to β-carotene and other carotenoids in the plastid via the methylerythritol phosphate (MEP) pathway [4]. Carotenoids are plastidic tetraterpenoids and are the essential building blocks for vitamin A and plant-protectant compounds such as lycopene in colored plant parts of fruits and vegetables [5,6]. Silencing the *PDS* gene via RNAi results in a visible “photo-bleached leaf” phenotype that has been instrumental in the study of plant functional genomics [2,3,7].

The efficiency of gene silencing is influenced by many factors, including the VIGS vector, the host plant, and the gene selected for targeting [1]. In addition, the bleached-leaf phenotype is more pronounced, when the antisense orientation of the construct is used to be compared to the sense orientation [8]. Although *PDS* gene expression is down-regulated in both sense and antisense orientations, the greater silencing of *PDS* is induced due to the production of subgenomic RNAs that complements the small interfering RNAs, resulting in a stronger phenotype [9]. The stronger phenotype correlates well with higher levels of accumulated phytoene and lower levels of xanthophylls and carotenes in citrus plants treated with antisense-oriented constructs [8]. Finally, because certain plant volatile organic compounds (VOCs) arise from the carotenoid biosynthetic pathway, the effects of the *PDS* suppression on the VOC profile of CTV-t*PDS* citrus were recently studied [10].

Plants emit volatile compounds for communication, to attract pollinators and in response to infestation and herbivory by insects [4,11]. Emission into the atmosphere can occur passively by diffusion through the cell membranes or through the disruption of the storage glands during herbivory or other physical damage to plant tissues. *Diaphorina citri* respond to both olfactory and visual cues when seeking a host plant and are attracted to the alterations in the leaf color and VOC profile of *PDS*-silenced *Citrus macrophylla* [10]. In that study, monoterpenes were increased, and sesquiterpenes were decreased, suggesting some correlation between the disruption of biosynthetic pathways and the altered VOCs profiles found in CTV-t*PDS* citrus [10]. However, in that study, volatiles were collected from *PDS*-silenced *C. macrophylla* without discrimination against the degree of the photobleaching phenotype [10].

Hundreds of plant-emitted VOCs can be classified into 10 major groups stemming from four main biosynthetic pathways: (1) green leaf volatiles and jasmonates, arising from fatty acids in the acetate/lipoxygenase pathway; (2) cinnamates, benzoates, and methyl chavicol from the shikimate pathway; (3) sesquiterpenes (C_15_) derived from the mevalonic acid (MVA) pathway in the cytosol; and (4) monoterpenes (C_10_), diterpenes (C_20_), and apocarotenoids arising from the plastidic MEP pathway [4,12]. Even so, given the large diversity of volatile compounds synthesized in citrus, the specific pathways and steps limiting their biosynthesis remain to be ascertained. Therefore, we hypothesized that the VOC profile of the CTV-t*PDS* citrus would be altered in a gradient-based manner according to the degree of the photobleached-leaf phenotype. 

## 2. Results 

### 2.1. The Photobleaching Phenotype Is Correlated to the CTV Titer

**Figure 1:**
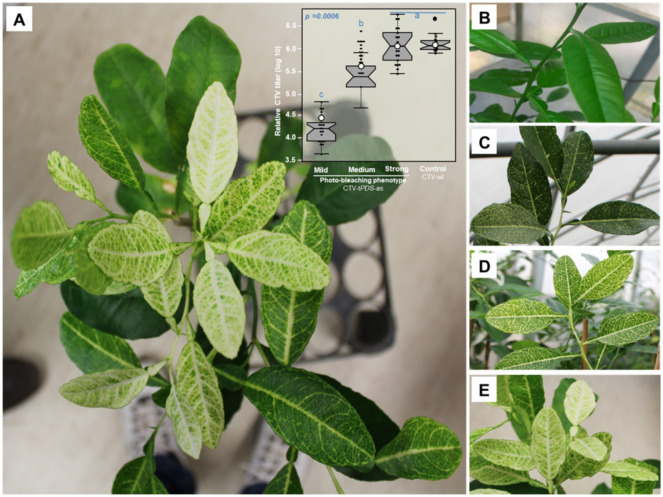
Silencing phytoene desaturase (PDS) causes a photobleaching phenotype in citrus plants: (**A**) photo showing the bleaching phenotype was not homogenous within the tree due to the unequal distribution of the *Citrus tristeza virus* (CTV)-t*PDS*-as vector. Note the different degrees of phenotype. The phenotype strength was correlated with the CTV titer (upper right corner in (**A**)); (**B**) photo showing the control plant (CTV-wt) did not show a photobleaching phenotype; (**C**) photo showing the mild phenotype; (**D**) photo showing the medium phenotype; (**E**) photo showing the strong phenotype.

In this study, we used PDS-silenced *C. macrophylla* using an antisense orientation (CTV-t*PDS*-as). We noticed that the photobleached phenotype in the *PDS*-silenced citrus was not homogenous within the tree (Figure 1A). We selected three degrees of the phenotype (i.e., mild, medium, and strong) in addition to the control (CTV-wt) (Figure 1B–E). The qPCR showed a positive correlation between the CTV titer and the strength of phenotype in the *PDS*-silenced plant. However, in the control plant that was inoculated with the empty vector (CTV-wt), the virus titer was as high as the strong phenotype of the *PDS*-silenced plant but did not show any photobleaching (Figure 1A, the upper right). 

### 2.2. VOCs Released from C. Macrophylla

**Figure 2:**
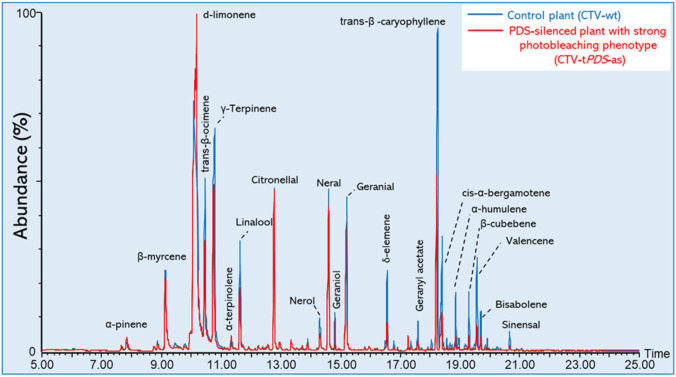
Overlayed total ion chromatograms (TICs) of released volatiles from citrus leaves separated by gas chromatography-mass spectrometry (GC-MS) analysis. The green peaks indicate released leaf volatiles from the control plants (CTV-wt), while red peaks indicate released leaf volatiles from PDS-silenced plants (CTV-t*PDS*-as) with a strong photobleaching phenotype.

To test the relationship between the carotenoid pathway and the production of VOCs, we selected leaves with varying degrees of *PDS* gene expression (photobleaching phenotype) and collected their released volatiles using solid phase microextraction (SPME) followed by gas chromatography-mass spectrometry (GC-MS) (Figure 2).

We collected the released volatiles from the mature, fully expanded leaves of *C. macrophylla* plants inoculated with CTV-t*PDS*-as categorized with having a “mild”, “medium”, or “strong” phenotype and compared them to emissions from *C. macrophylla* inoculated with the empty vector CTV-wt as the control plants (Figure 1B–E).

Overall, 47 volatile compounds were detected, identified mainly with authentic standards and quantified using the peak areas (Table 1). The compounds were classified into 12 VOC classes including 13 monoterpenes, 11 sesquiterpenes, six monoterpene alcohols, five monoterpene aldehydes, three monoterpene esters, three sesquiterpenes alcohols, and six compounds from other classes. The biosynthetic pathway associated with each compound class is indicated in Table 1.

### 2.3. Prencipal Component Analysis (PCA)

**Figure 3:**
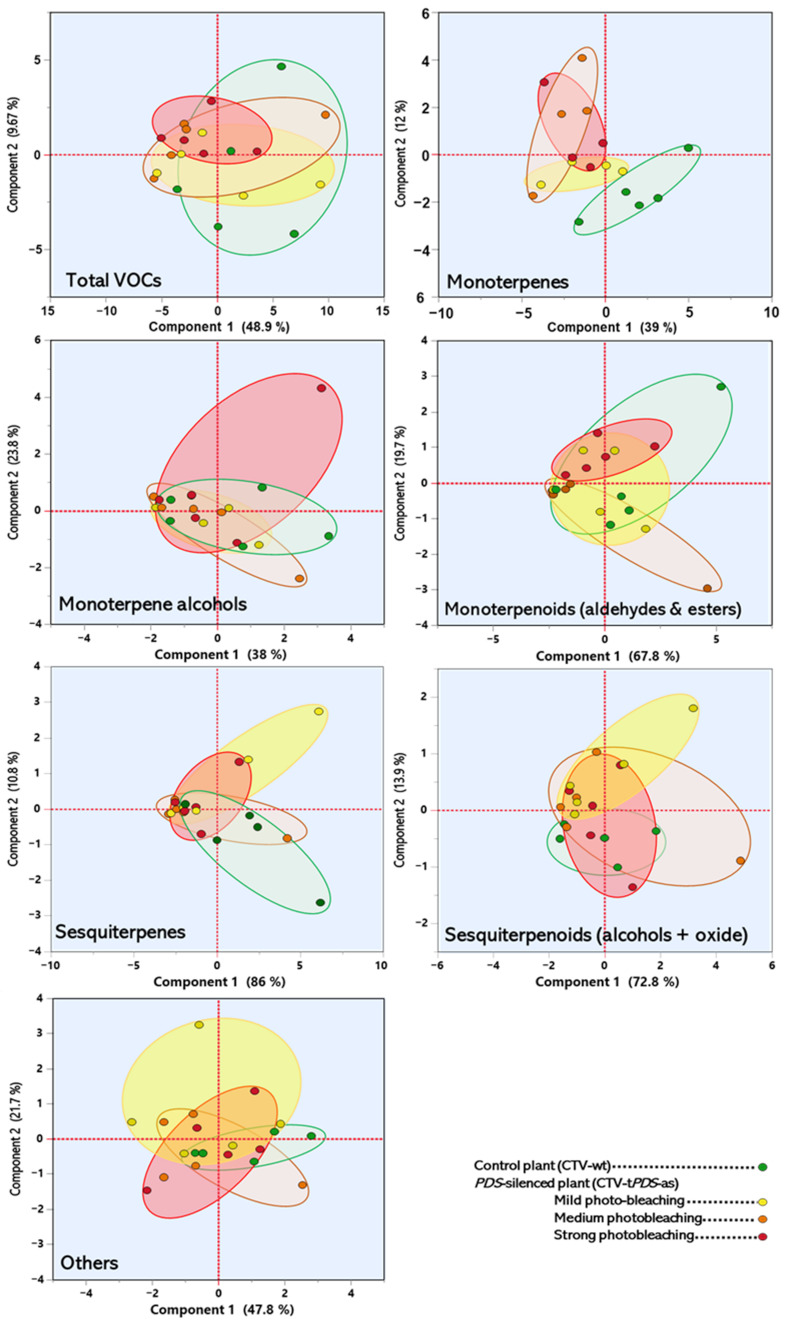
Principal component analysis of the VOCs released from the leaves of *C. macrophylla* with different degrees of photobleaching using total VOC and individual classes. For the members of each class, see Table 1.

To discriminate the effects of the silencing of *PDS* with different degrees of photobleaching on the production of VOCs, PCA was performed using all detected compounds or only main classes. When all compounds were used, no clear separation was observed (Figure 3). Similar results were observed when individual groups were used. Only the PCA performed with monoterpenes showed a clear separation for the three degrees of photobleaching from the control leaves (Figure 3). This finding suggested that monoterpenes is the main class of volatile that is affected with the reduction in the carotenoid pathway.

### 2.4. Hierarchical Clustering, Heat Map, and Simple Linear Regression

**Figure 4:**
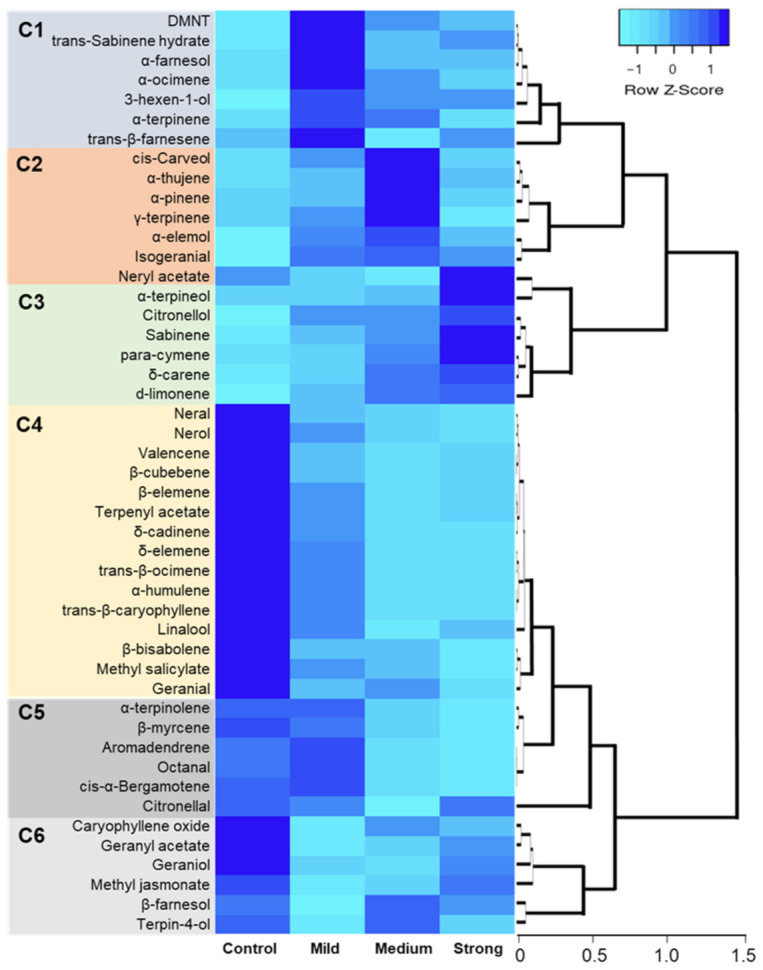
Two-way hierarchical cluster analysis and heat map of the VOCs released leaves from *C. macrophylla* with different degrees of photobleaching. Clusters 1–6 represent groups of compounds with a similar response to the reduction of carotenoids. Rows represent compounds, while columns represent treatments (photobleaching degrees). Cells are the mean peak area of each compound (*n* ranges from 6 to 8).

The two ways hierarchical cluster analysis and heat map diagram of the volatiles released from citrus leaves with a gradient degree of the PDS leaf phenotype are shown in Figure 4.

Cluster 1 (C1) depicted those compounds associated with the mild phenotype. They included 4,8-dimethyl-1,3,7-nonatriene (DMNT), α-terpinene, *trans*-sabinene hydrate, and α-ocimene (derived from the MEP pathway), *trans*-β-farnesene and α-farnesol that are derived from the MVA pathway, and the green leaf volatile 3-hexen-1-ol derived from the lipoxygenase (LOX) pathway. Cluster 2 (C2) compounds were associated with the medium grade of phenotype and included α-thujene, α-pinene, γ-terpinene, isogeranial, *cis*-carveol, and α-elemol. All, except α-elemol, were derived from the MEP pathway. Cluster 3 (C3) and cluster 4 (C4) were associated with the VOCs that correlated well (positively or negatively) with the increasing PDS phenotype strength. C3 compounds were associated with the strongest PDS phenotype and increased in the concentration with the increasing phenotype. Using simple linear regression analysis allowed us to study relationships between two continuous (quantitative) variables. In our case, the two variables were the degree of the photobleaching phenotype (carotenoid content) and the quantity of a VOC. The simple linear regression revealed the correlation between carotenoids and volatile production, suggesting a possible connection.

Simple liner regression confirmed this correlation (Table 1). These included sabinene (*p* = 0.0239), δ-carene (*p* = 0.0046), *para*-cymene (*p* = 0.0256), d-limonene (*p* = 0.0296), α-terpineol, citronellol, and neryl acetate, and all were from the MEP pathway. Cluster 4 consisted of 15 compounds, the largest of the clusters generated by the hierarchical clustering analysis. The compounds in C4 were highest in the control plants (CTV-wt), and their concentrations generally decreased with the increasing phenotype. Simple liner regression confirmed this correlation (Table 1). These compounds included *trans*-β-ocimene (*p* = 0.0303), linalool, methyl salicylate (*p* = 0.0363), nerol, neral, geranial, terpenyl acetate, δ-elemene (*p* = 0.0405), β-elemene, *trans*-β-caryophyllene, β-cubebene, α-humulene, valencene, β-bisabolene, and δ-cadinene. About half (8 of 15) C4 compounds were from the MVA pathway. Cluster 5 (C5) was correlated with the compounds that increased slightly in the mild phenotype compared to the control and then decreased with the increasing phenotype. These included β-myrcene (*p* = 0.015), octanal, α-terpinolene (*p* = 0.045), and citronellal from the MEP pathway and aromadendrene and *cis*-α-bergamotene from the MVA pathway. Finally, C6 represented compounds that were initially reduced in the mild phenotype and then recovered with increasing phenotype or which had a mixed response to the PDS phenotype. The C6 group included compounds from several biosynthetic pathways: terpin-4-ol, geraniol, and geranyl acetate (MEP); caryophyllene oxide and β-farnesol (MVA); and methyl jasmonate (shikimate). None were well correlated with the PDS phenotype gradient (Figure 4).

Thus, overall, the VOCs detected via SPME/GC-MS with statistically significant changes in the emission quantity with respect to the degree of the leaf phenotype using linear regression analysis included seven monoterpenes, one sesquiterpene (δ-elemene), and one benzenoid (methyl salicylate). The monoterpenes with strong correlations to phenotype included sabinene, β-myrcene, δ-carene, *para*-cymene, d-limonene, *trans*-β-ocimene, and α-terpinolene.

## 3. Discussion

PDS gene is widely used as a reporter gene for the virus-induced gene silencing technique, because the knockdown of *PDS* creates a specific photobleaching phenotype as a result of reduced carotenoid production [13,14,15,16]. We utilized CTV to construct a gene-silencing vector, and *PDS* was used as a reporter gene to demonstrate its efficiency [1]. In addition, we previously showed that the efficacy of silencing was higher when we used the antisense orientation of truncated *PDS* [8]. Furthermore, we showed that the augmented silencing effect observed after using the antisense orientation was caused by additional subgenomic RNA that was available as a supplemental source for complementary sequences, thereby resulting in more RNA inference and a subsequent increase in the photobleaching phenotype [8]. Silencing *PDS* in citrus resulted in the alterations of both carotenoids and chlorophyll pigments, indicating that carotenoids play a photoprotective role for chlorophyll and that when reducing carotenoids by *PDS* silencing, chlorophyll pigments may be more exposed to light-causing degradation [8].

Interestingly, *PDS*-silenced citrus plants were more attractive to *Diaphorina citri*, the psyllid vector of “*Candidatus* Liberibacter asiaticus”, the putative bacterial pathogen of huanglongbing in citrus, suggesting that these plants could potentially be used as trap crops [10]. The preference assay, electrical penetration graph, and chemical analysis indicated that these plants provide gustatory, visual, and olfactory cues that attract *D. citri* [10]. *PDS*-silenced plants exhibited an enriched metabolite content of the phloem sap, which offered appropriate gustatory cues that influenced probing/feeding behavior, while the bleaching phenotype on leaves provided a sufficient close-range visual attractant to stimulate *D. citri* landing [10]. Alternately, the olfactory cues that attracted *D. citri* to *PDS*-silenced plants could stem from the alteration in the biosynthesis and release of VOCs from leaves [10], demonstrating an interplay between the carotenoid and VOCs pathways.

The photobleaching in *PDS*-silenced citrus plants is not homogeneous within the tree due to the unequal distribution of CTV particles and the co-localization of the RNA inference with the CTV particles [8]. In the current study, we took advantage of this phenomenon to investigate the correlation between the carotenoid pathway and VOCs biosynthesis by using different degrees of photobeaching and the in vivo collection of VOCs. Although many VOCs were altered in *PDS*-silenced citrus plants, most VOCs that showed a strong correlation (statistically significant) with the photobleaching degree were monoterpenes belonging to the MEP pathway. These VOCs included sabinene, β-myrcene, δ-carene, *para*-cymene, d-limonene, *trans*-β-ocimene, α-terpinolene, citronellol, and geraniol. It is worth mentioning that the carotenoid and MEP biosynthetic pathways are localized in the plastid [4]. This co-localization and the gradient response of MEP monoterpenes with a contemporaneous reduction of carotenoids suggest an alternative route from carotenoids for theses volatiles. In support of our findings, Lewinsohn, et al. [17] showed that a yellow-fleshed tomato (r) mutant and water melon (early moonbeam), which carry nonfunctional phyotene synthase gene (*psy*1), were almost absent of geranial and several volatiles derivatized from norisoprenoid. This previous study suggested an alternative route to geranial from the carotenoids neurosporene, lycopene, and δ-carotene and confirmed that degraded carotenoids provided a substrate for many aroma compounds in tomato and watermelon [17].

Collectively, current findings strongly suggested the presence of a correlation between the carotenoid content and monoterpenes. More in-depth investigations are needed to increase our understanding regarding the correlation between the carotenoid pathway and the MEP pathway, which is responsible for the production monoterpenes, and their biosynthetic regulation in plants.

## 4. Materials and Methods

### 4.1. Production of CTV-wt and CTV-tPDS Plants

*PDS*-silenced citrus plants were produced in our previous study [8]. Briefly, the infectious cDNA clone of CTV (isolate T36; GenBank accession no. AY170468) in the binary vector pCAMBIA-1380 was used as a base plasmid, and a truncated *PDS* gene in its antisense orientation was used for engineering the construct (CTV-t*PDS*-as) [8]. CTV-t*PDS*-as and CTV-wt were agroinfiltrated into *Nicotiana benthamiana* to propagate the virions. CTV virions were isolated from *N. benthamiana* and inoculated into *Citrus macrophylla* seedlings [1]. The CTV titer was estimated using qPCR as described by Killiny [9].

### 4.2. Plant Maintenance

Experimental plants were about 12 months old and were maintained at 28 ± 2 °C, with 65 ± 5% relative humidity (RH), and a 16-h light/8-h dark photoperiod in a USDA-APHIS-approved secured greenhouse (Citrus Research and Education Center, University of Florida, Lake Alfred, FL, USA). Plants were irrigated twice weekly, fertilized with water soluble 20-10-20 NPK weekly (Peter’s Florida Special, Allentown, PA, USA) and once quarterly with a 16-8-10 NPK slow-release fertilizer (Harrell’s, Lakeland, FL, USA).

### 4.3. In Vivo VOC Collection

**Figure 5:**
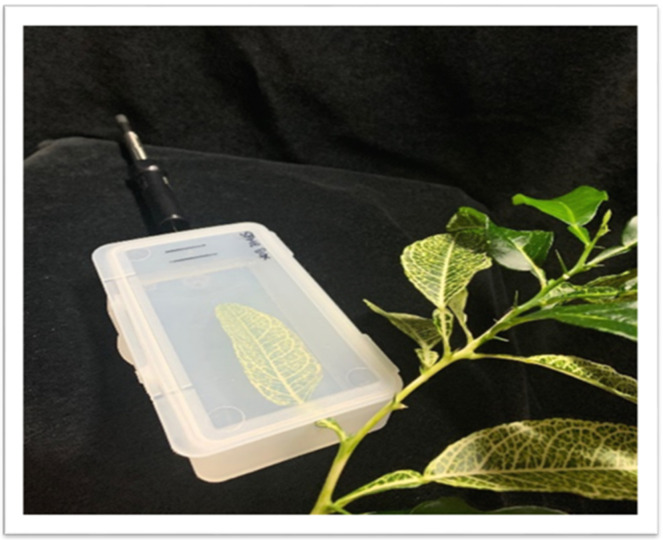
Collection of the released VOCs from citrus leaves using the overhead space. Leaf was placed lying flat inside a clear plastic box with a notch cut out at one end for the stem and a pinhole at the other end for fiber entry.

Released VOCs were collected from intact plant leaves using SPME and were analyzed by GC-MS. The SPME fiber was a mixed polarity fiber (#57328U, Supelco, Bellefonte, PA, USA), as previously reported for the in vivo collection of citrus leaf volatiles [10,18]. This SPME fiber was chosen for its capacity to trap a broad range of volatile compounds [18]. The collection device was modified from that previously reported to allow for VOC collection from the leaf in a more natural position (Figure 5). Each leaf was placed lying flat (adaxial epidermis up) inside a clear plastic clamshell box about the size of a cassette tape holder (120 mm long × 75 mm wide × 10 mm deep) with a notch cut out at one end for the stem and a pinhole at the other end for fiber entry. After the box was closed around the leaf, it was sealed carefully with a strip of Parafilm^®^ M. As previously reported, we collected the released volatiles at 27 °C for 2 h from the static headspace above each enclosed leaf. 

### 4.4. GS

After the collection period, the fiber was desorbed into the GC-MS inlet for 5 min at 250 °C splitlessly, and the volatiles were separated on an Elite-5 column (30 mL × 0.25 mm I.D. × 0.25 µm film thickness, Perkin Elmer, Waltham, MA, USA) with ultrahigh purity helium as the carrier gas. The chromatographic conditions, peak area normalization, and compound identification procedures were identical to those previously reported in Killiny and Jones [18].

### 4.5. Statistical Analysis

Statistical analyses were performed using JMP version 9.0 (SAS Institute Inc., Cary, NC, USA). Data were normally distributed. Each treatment (a degree of photobleaching) was composed of five biological replicates. Simple linear regressions were conducted to elucidate the correlations between the detected volatiles and the degree of photobleaching in the CTV-t*PDS*-as plants. The two-way Hierarchical Cluster Analysis (HCA) and the heat map of released volatiles from C. macrophylla leaves with different degrees of photobleaching were generated using the means of the data matrices based on the Ward’s minimum variance method. 

## 5. Conclusions

Volatiles were synthetized through many biosynthetic pathways in plastid, mitochondria, and cytosol and released through leaf openings such as stomata. In addition to the MEP pathway, plastid hosted many other pathways including chlorophyll, carotenoids, and shikimic acid pathways. The MEP pathway was responsible for the production of isoprene, monoterpenes, and diterpenes. Diterpenes fed to the homoterpenes and carotenoids pathways. Silencing PDS caused photobleaching phenotypes (reduction of carotenoids contents) in citrus leaves. Monoterpenes released from citrus leaves were significantly correlated with the degree of photobleaching. These findings suggested a relation between carotenoid and MEP pathways. More investigations are needed to reveal this connection.

## Figures and Tables

**Table 1:** Volatile organic compounds (VOCs) from hexane-extracted leaves of *C. macrophylla* and their GC-MS chromatographic parameters, terpene class and biosynthetic pathway. Simple linear regression was performed using the peak areas of each compound in *C. macrophylla* leaf extracts with a gradient of the photobleaching (control, mild, medium, and strong) phenotype.

Compound	RT	Compound	Identifier ions (*m/z*)	RI	Chemical formula	Pathway	Terpene class	SLR equation	*p*-value
1	6.00	3-Hexen-1-ol ^a^	67, 82, (100)	838	C_6_H_12_O	LOX	Green leaf volatile	Y = 0.6615007 + 1.5039 × 10^−6^ × X	0.6371
2	7.66	α-thujene ^a^	93, (136)	900	C_10_H_16_	MEP	Monoterpene	Y = −1.124171 + 4.2505 × 10^−8^ × X	0.5255
3	7.83	α-pinene ^b^	77, (136)	946	C_10_H_16_	MEP	Monoterpene	Y = 0.5354597 + 5.293 × 10^−9^ × X	0.8134
4	8.74	sabinene ^b^	93, (136)	962	C_10_H_16_	MEP	Monoterpene	Y = −2.043718 + 6.3962 × 10^−8^ × X	0.0239
5	9.12	β-myrcene ^b^	77, (136)	989	C_10_H_16_	MEP	Monoterpene	Y = 5.4066127 − 5.6089× 10^−9^ × X	0.0151
6	9.40	octanal ^b^	56, 84, (128)	1008	C_8_H_16_O	LOX	Aliphatic aldehyde	Y = 9.7890063 − 1.1892 × 10^−7^ × X	0.1339
7	9.58	δ-carene ^b^	93, (136)	1009	C_10_H_16_	MEP	Monoterpene	Y = −1.024644 + 4.9396 × 10^−8^ × X	0.0046
8	9.78	α-terpinene ^b^	121, (136)	1018	C_10_H_16_	MEP	Monoterpene	Y = 1.6879193 − 3.3425 × 10^−9^ × X	0.9665
9	9.95	*para*-cymene ^b^	119, (134)	1026	C_10_H_14_	MEP	Monoterpene	Y = −0.309639 + 9.4336 × 10^−9^ × X	0.0256
10	10.08	d-limonene ^b^	68, (136)	1038	C_10_H_16_	MEP	Monoterpene	Y = −5.256629 + 1.2465 × 10^−9^ × X	0.0296
11	10.42	*trans*-β-ocimene ^b^	107, (136)	1055	C_10_H_16_	MEP	Monoterpene	Y = 5.4322736 − 6.2774 × 10^−9^ × X	0.0303
12	10.72	γ-terpinene ^b^	93, 121, (136)	1071	C_10_H_16_	MEP	Monoterpene	Y = 1.6124691 − 7.942 × 10^−11^ × X	0.9824
13	10.99	*trans-*sabinene hydrate ^a^	93, (154)	1081	C_10_H_18_O	MEP	Monoterpene	Y = 0.9934156 + 4.3591 × 10^−8^ × X	0.5838
14	11.34	α-terpinolene ^b^	93, (136)	1101	C_10_H_16_	MEP	Monoterpene	Y = 5.3820149 − 3.3693 × 10^−8^ × X	0.0450
15	11.62	linalool ^b^	71, 80, (154)	1114	C_10_H_18_O	MEP	Monoterpene alcohol	Y = 5.8499642 − 1.2174 × 10^−8^ × X	0.1903
16	11.92	DMNT ^b^	135, (150)	1131	C_11_H_18_	MVA	Homoterpene	Y = 1.3459995 + 8.853 × 10^−10^ × X	0.9179
17	12.25	α-ocimene ^a^	93, (136)	1152	C_10_H_16_	MEP	Monoterpene	Y = 2.0591518 − 1.3416 × 10^−8^ × X	0.8609
18	12.75	citronellal ^b^	69, (154)	1178	C_10_H_18_O	MEP	Monoterpene aldehyde	Y = 2.8260106 − 9.765 × 10^−10^ × X	0.6888
19	13.44	isogeranial ^a^	81, (152)	1211	C_10_H_16_O	MEP	Monoterpene aldehyde	Y = −1.524911 + 6.9897 × 10^−8^ × X	0.7376
20	13.55	Terpin-4-ol ^b^	71, 111, (154)	1225	C_10_H_18_O	MEP	Monoterpene alcohol	Y = 3.0069503 − 1.3479 × 10^−7^ × X	0.7797
21	13.71	methyl salicylate ^b^	120, (152)	1228	C_8_H_8_O_3_	Shikimate	Benzenoid	Y = 5.6708351 − 1.4192 × 10^−7^ × X	**0.0363**
22	13.82	α-terpineol ^b^	59, (136)	1233	C_10_H_18_O	MEP	Monoterpene aldehyde	Y = 0.5691447 + 1.0031 × 10^−7^ × X	0.2207
23	14.31	nerol ^b^	69, (154)	1264	C_10_H_18_O	MEP	Monoterpene alcohol	Y = 4.1787856 − 1.3466 × 10^−7^ × X	0.0885
24	14.35	citronellol ^b^	69, (156)	1267	C_10_H_20_O	MEP	Monoterpene alcohol	Y = −2.214841 + 8.4327 × 10^−8^ × X	**0.0467**
25	14.42	*cis*-carveol ^b^	109, (152)	1275	C_10_H_16_O	MEP	Monoterpene alcohol	Y = 0.6619045 + 4.0657 × 10^−8^ × X	0.6612
26	14.56	neral ^b^	69, (152)	1280	C_10_H_16_O	MEP	Monoterpene aldehyde	Y = 4.2283559 − 3.3036 × 10^−9^ × X	0.1167
27	14.86	geraniol ^b^	69, (154)	1292	C_10_H_18_O	MEP	Monoterpene alcohol	Y = 2.1508954 − 2.2447 × 10^−8^ × X	**0.4888**
28	15.16	geranial ^b^	69, (152)	1308	C_10_H_16_O	MEP	Monoterpene aldehyde	Y = 4.2872926 − 2.9944 × 10^−9^ × X	0.1342
29	16.56	δ-elemene ^a^	121, (204)	1360	C_15_H_24_	MVA	Sesquiterpene	Y = 3.5948159 − 1.1139 × 10^−8^ × X	**0.0405**
30	16.75	terpenyl acetate ^b^	121, 136, (181)	1366	C_12_H_20_O_2_	MEP	Monoterpene Ester	Y = 4.5742907 − 1.2065 × 10^−7^ × X	0.1008
31	16.91	neryl acetate ^b^	69, (196)	1373	C_12_H_20_O_2_	MEP	Monoterpene Ester	Y = 0.6446098 + 8.1427 × 10^−8^ × X	0.5523
32	17.30	geranyl acetate ^b^	69, (196)	1378	C_12_H_20_O_2_	MEP	Monoterpene Ester	Y = 3.2687338 − 3.1068 × 10^−8^ × X	0.5486
33	17.59	β-elemene ^b^	93, (189)	1384	C_15_H_24_	MVA	Sesquiterpene	Y = 3.7802501 − 3.4001 × 10^−8^ × X	0.1234
34	18.22	*trans*-β-caryophyllene ^b^	133, (204)	1461	C_15_H_24_	MVA	Sesquiterpene	Y = 4.7685504 − 2.6421 × 10^−9^ × X	0.0789
35	18.35	aromadendrene ^a^	161, (204)	1476	C_15_H_24_	MVA	Sesquiterpene	Y = 4.4307055 − 2.1659 × 10^−8^ × X	0.1442
36	18.40	*cis*-α-bergamotene ^a^	119, (204)	1482	C_15_H_24_	MVA	Sesquiterpene	Y = 4.4877318 − 8.7324 × 10^−9^ × X	0.1192
37	18.77	*trans*-β-farnesene ^b^	93, (204)	1505	C_15_H_24_	MVA	Sesquiterpene	Y = 1.9219621 − 4.4732 × 10^−8^ × X	0.8137
38	18.85	α-humulene ^b^	93, (204)	1511	C_15_H_24_	MVA	Sesquiterpene	Y = 4.0697399 − 2.3463 × 10^−8^ × X	0.0814
39	19.30	β-cubebene ^a^	161, (204)	1526	C_15_H_24_	MVA	Sesquiterpene	Y = 3.184197 − 1.9774 × 10^−8^ × X	0.1491
40	19.55	valencene ^b^	189, (204)	1535	C_15_H_24_	MVA	Sesquiterpene	Y = 3.2005192 − 1.1617 × 10^−8^ × X	0.1518
41	19.69	β-bisabolene ^b^	93, (204)	1541	C_15_H_24_	MVA	Sesquiterpene	Y = 4.3461062 − 3.8601 × 10^−8^ × X	0.0665
42	19.91	δ-cadinene ^a^	93, (204)	1549	C_15_H_24_	MVA	Sesquiterpene	Y = 4.5764693 − 1.2446 × 10^−7^ × X	0.0784
43	20.44	α-elemol ^a^	59, 93, (204)	1563	C_15_H_26_O	MVA	Sesquiterpene alcohol	Y = −2.631255 + 1.2806 × 10^−6^ × X	0.5189
44	21.07	caryophyllene oxide ^b^	79, 93, (220)	1589	C_15_H_24_O	MVA	Sesquiterpenoid	Y = 4.5729864 − 3.0942 × 10^−7^ × X	0.5161
45	21.89	methyl jasmonate ^b^	84, 151, (224)	1665	C_13_H_20_O_3_	LOX	Jasmonates	Y = 1.8891837 − 1.0704 × 10^−7^ × X	0.9031
46	22.69	β-farnesol ^b^	69, (222)	1725	C_15_H_26_O	MVA	Sesquiterpene alcohol	Y = 0.5641273 + 5.0138 × 10^−7^ × X	0.9155
47	23.19	α-farnesol ^b^	69, (222)	1740	C_15_H_26_O	MVA	Sesquiterpene alcohol	Y = 1.5524536 − 1.7063 × 10^−7^ × X	0.9731

^a^ Tentatively identified by an linear retention index (LRI) and a library matching score of >700. ^b^ Compound confirmed by an authentic reference standard. Bold *p*-values indicate significance (*p* < 0.05). RT: retention time; RI: retention index.

## Data Availability

All data are contained within the article.
